# CFX: Contention-Free Channel Access for IEEE 802.11ax

**DOI:** 10.3390/s22239114

**Published:** 2022-11-24

**Authors:** Kyu-haeng Lee, Daehee Kim

**Affiliations:** 1 Department of Mobile Systems Engineering, Dankook University, Yongin-si 16890, Gyeonggi-do, Republic of Korea; 2 Department of Internet of Things, Soonchunhyang University, Asan-si 31538, Chungcheongnam-do, Republic of Korea

**Keywords:** 802.11ax, reinforcement learning, OFDMA, MAC

## Abstract

Orthogonal frequency-division multiple access (OFDMA) has attracted great attention as a key technology for uplink enhancement for Wi-Fi, since it can effectively reduce network congestion and channel access delay. Unfortunately, the traditional random access protocol of Wi-Fi seldom allows these benefits to be achieved, especially in dense network environments, as the access point (AP) rarely gains the channel access needed to trigger OFDMA uplink transmissions due to severe frame collisions. To address this problem, we propose a new channel access scheme called Contention-Free Channel Access for 802.11ax (CFX). In the proposed scheme, users can access the channel without contention, since they are guaranteed a transmission opportunity immediately after another user’s transmission. To realize CFX on top of the existing Buffer Status Report/BSR Poll (BSR/BSRP) exchange protocol of 802.11ax, we develop an additional scheme based on shared channel access that helps the AP to obtain the buffer status of users and manage a contention-free channel access schedule. In addition, in order to appropriately utilize the savings from the reduced frame collisions, we conduct sum throughput maximization using an actor-critic proximal policy optimization (PPO)-based deep reinforcement learning approach. The results of an extensive evaluation show that CFX not only significantly improves the uplink performance of Wi-Fi in terms of throughput and channel access delay but can also dynamically adjust the parameters in response to changes in the network status.

## 1. Introduction

With the recent introduction of orthogonal frequency-division multiple access (OFDMA) to IEEE 802.11ax [1], the latest Wi-Fi standard, the demand for significant improvements in the uplink of Wi-Fi systems is increasing. OFMDA divides the whole channel bandwidth into several small subchannels called resource units (RUs) and allows multiple wireless nodes to communicate with each other concurrently, thus effectively reducing the network congestion and preamble overhead, increasing the system throughput, and shortening the channel access delay. According to a study by Qualcomm [2], exploiting the features of OFDMA can deliver a reduction in uplink latency of up to 63% in a busy network scenario with 16 Wi-Fi connections. For this reason, OFDMA is regarded as a key technology of Wi-Fi in terms of supporting a variety of high-demand applications, such as peer-to-peer computing, streaming services, IoT systems, and cloud applications [3,4,5].

Although OFDMA has the potential to deliver high performance gains, the well-known frame collision problem in a Wi-Fi environment unfortunately makes it almost impossible to use, especially in dense network environments [6]. To enable OFDMA in Wi-Fi, the 802.11ax standard states that the access point (AP) controls all OFDMA transmissions in the uplink direction. More specifically, the AP triggers the actual uplink OFDMA transmission for user devices (hereafter referred to as users) by broadcasting a trigger frame (TF), which implies that an OFDMA transmission can be activated only when the AP wins the channel contention. Since channel access in Wi-Fi basically operates in a fully distributed manner through distributed coordination function (DCF), frame collisions among the multiple transmitting devices are inevitable. In several actual products, some control frames which play an essential role in Wi-Fi (e.g., beacon frames) are given a higher transmission priority to avoid being transmitted too late [7,8], but this does not mean that the AP is free from control frame collisions [9,10,11]. A rough calculation using Bianchi’s model [12] indicates that the probability of a collision can rise to 10% (with a corresponding access probability τ=0.026 (In this calculation, the AP is assumed to have the same access probability as non-AP users.)) when there are 20 devices. These high collision and low access probabilities imply that the AP has little opportunity to invoke an OFDMA transmission.

There have been numerous efforts to resolve this issue. In the 802.11 family, several control frames and an auxiliary MAC protocol are already included for this purpose. For example, short-sized control frames such as Request To Send/Clear To Send (RTS/CTS) frames may be exchanged before actual data transmission begins to reduce the time consumed by collisions. Point coordination function (PCF) is an alternative to DCF that allows the AP to schedule and control uplink transmissions to avoid unnecessary frame collisions. These methods, however, involve a non-negligible protocol overhead, and if its impact is not properly considered, then the actual performance gain may be severely limited. For this reason, they were included as optional features in the standards, and in particular, PCF is rarely implemented in actual products as it is obsolete now.

A number of attempts have been made to exploit various factors affecting the OFDMA performance in 802.11ax, such as the maximum aggregate frame size, the number of antennas at the AP [13], channel aggregation [14], and the number of random access RUs [15]. Unfortunately, while most of these approaches require significant changes to the existing MAC protocol, they may quickly become less effective in networks containing users of the legacy MAC protocol. Recently, deep reinforcement learning approaches have been widely applied to dynamically control the MAC parameters and increase channel utilization [16,17,18,19]. However, the unique features of 802.11ax, such as the TF-based protocol, are not well utilized in most of these schemes. In addition, they assume that each individual user needs to run a deep reinforcement learning module, which may be practically infeasible for resource-constrained devices.

To address the aforementioned issues, we propose a new scheme that provides contention-free (CF) channel access for IEEE 802.11ax called CFX. Our idea is straightforward: we allow users to access the channel without contention. Instead, they are guaranteed a transmission opportunity for a certain amount of time immediately after another user’s transmission has finished. CFX inherits the existing 802.11ax uplink OFDMA protocol, meaning that the proposed channel access scheme can be entirely managed by the AP. The AP performs scheduling for CF channel access based on a consideration of the buffer status of the users, and the users follow the AP’s guidance to transmit their frames. CFX is a DCF-friendly access scheme. Even if the channel is shared with legacy devices that use existing random access protocols, CFX does not harm these devices and instead improves their performance.

Although the idea underlying the proposed scheme may appear simple, several technical challenges need to be resolved to realize CFX. First, in order to effectively schedule CF channel access for users, the AP needs to keep tracking their demands for uplink transmissions. To achieve this, we fully exploit the Buffer Status Report/BSR Poll (BSR/BSRP) exchange protocol that was newly proposed in 802.11ax, and for ease of use, we develop an additional scheme called shared channel access. In this scheme, when a user gains an opportunity to access the channel, it does not transmit immediately but instead requests the AP to trigger an OFDMA uplink transmission to perform simultaneous transmissions with other users. As a result, our scheme not only helps the AP to effectively obtain the buffer status of each user but also allows the users to access the channel more frequently, thus reducing the channel access delay.

Ideally, the savings from the reduced probability of channel collision should be appropriately returned to all of the users in the network. This aspect should be carefully taken into account in the case of networks where CFX users and legacy users coexist, as incorrect resource allocation may cause unfairness between them. To ensure proper resource allocation, we optimize our CFX scheme. We formulate a sum throughput maximization problem under the constraint of minimum performance requirements for all types of users in terms of throughput and channel access delay. To solve the optimization problem effectively, we employ a deep reinforcement learning (DRL) approach in which we exploit an actor-critic-based Proximal Policy Optimization (PPO) learning algorithm to allow CFX to dynamically adjust its parameters to the varying network conditions.

To demonstrate the feasibility of the proposed scheme, we implement the key functionalities of CFX using the MATLAB deep learning framework [20]. Extensive evaluation results verify that CFX significantly lowers the frame collision probability (by up to 15%), meaning that both the legacy users and CFX users obtained higher performance gains. For both types of users, a throughput gain of more than 15% was achieved, and the channel access delays were reduced to 40% and 20%, respectively, in a network scenario with a heavy traffic load. It is also observed that the proposed PPO-based CFX optimization adjusted the parameters according to changes in the network status.

We summarize the contributions of this paper as follows:

- We propose a CF channel access scheme for 802.11ax, called CFX, that lowers the probability of frame collision by allowing users to access the channel without contention. CFX is a DCF-friendly scheme, and even if the channel is shared with legacy devices using DCF, CFX does not harm the legacy devices but instead improves their performance.
- We develop a shared channel access scheme to realize CFX. This method not only allows users to access the channel more frequently but also allows the AP to track the buffer status of each user effectively.
- We conduct CFX optimization. We formulate a sum throughput maximization problem under the constraints that the performance requirements for all users should be met. In particular, we adopt an actor-critic-based PPO to solve the optimization problem effectively.
- We verify the feasibility of the proposed scheme through extensive evaluations using the MATLAB deep learning framework. The results of this evaluation show that CFX significantly lowers the frame collision probability, and as a result, both the CFX users and the legacy users obtain performance gains in terms of throughput and channel access delay. It is also observed that the reinforcement learning module of CFX can suitably tune the parameters according to changes in the network status.

The remainder of this paper is organized as follows. Section 2 summarizes the results of prior research related to this work. We introduce the system model used in this paper in Section 3 and describe the proposed scheme in Section 4. Section 5 presents the results of a performance evaluation. Finally, the paper is concluded in Section 6.

## 2. Related Works

With the recent introduction of OFDMA into 802.11ax [1], the optimal allocation of OFDMA resources under different Wi-Fi scenarios has become a focus of interest for researchers. Karthik et al. controlled the contention window value and the maximum number of RUs that could be allocated to each user [21]. Dovelos et al. used Lyapunov optimization techniques to solve the uplink OFDMA resource allocation problem under average rate and power constraints [22], whereas Wang et al. investigated the user assignment problem with the goal of maximizing the sum rate [23]. Bankov et al. compared the performance of uplink OFDMA scheduling under different policies, such as max-rate, proportional fair and shortest remaining processing time [24].

The aforementioned OFDMA resource allocation schemes have frequently been discussed in numerous studies of other OFDMA-based systems, such as cellular networks. In contrast, the following proposals focus more on the distinct features of 802.11ax. In order to combine OFDMA with the existing random access protocol of Wi-Fi (i.e., DCF), 802.11ax allocates a certain proportion of the total available RUs to newly joined users and BSR frame transmissions, and users need to access these RUs (called random access RUs) in a random manner in the frequency domain. This mechanism is called Uplink OFDMA Random Access (UORA). As expected, there is a trade-off in terms of the proportion of random access RUs allocated; with a larger number of random access RUs, the AP can receive buffer status reports from more users, but the number of RUs available for OFDMA scheduling decreases, resulting in a lower actual OFDMA data transmission capacity. Lanante and Kotagiri et al. studied the impact of the number of random access RUs in uplink OFDMA scenarios [15,25]. For practical use cases involving UORA, 802.11ax adds two contention window parameters: the minimum OFDMA contention window (OCWmin) and maximum OFDMA contention window (OCWmax). There are several works that have designed models to analyze the performance of 802.11ax as a function of these parameters [26,27,28], and various other features of Wi-Fi have also been used to achieve better 802.11ax performance, such as the maximum A-MPDU size, the number of antennas at the AP [13], the EDCA parameter set [29], and channel aggregation [14]. Lee proposed the use of uplink OFDMA to achieve optimal downlink user selection in networks where 802.11ax and non-802.11ax coexist [30]. Kim et al. proposed a scheduling method based on the transmission delay of users for uplink multi-user transmissions in 802.11ax networks [31]. These methods are basically complementary to our scheme, which means that we can employ them in CFX. Note that in this work, we focus more on how to make the AP invoke OFMDA uplink transmissions more frequently than on how to improve OFDMA RU resource allocation.

Recently, diverse machine learning techniques have begun to be utilized to improve the performance of Wi-Fi networks in regard to various aspects of the system, such as access [16,17,18,19], channel selection [14], rate adaptation [32,33] and RU selection [15]. Han et al. used PPO-based reinforcement learning for channel aggregation [14], while Kotagiri et al. applied a DQN-based approach for random access RU selection with the objective of reducing the collision rate [15]. Chen et al. proposed a reinforcement learning-based rate adaption scheme for 802.11ac [34], the Wi-Fi standard prior to 802.11ax. They modeled the rate adaptation problem as a 3D maze problem by taking into account the three features that can affect the choice of data rate: Modulation and Coding Scheme (MCS), multiple-input multiple-output (MIMO) and bandwidth [32].

DRL has also been used to control the MAC parameters and enhance channel utilization. Wu et al. used Q-learning to adaptively adjust the length of the contention period in response to the current traffic rate in the network [16]. A centralized contention window optimization scheme (CCOD) using deep reinforcement learning was proposed by Wydmański et al. [17]. In CCOD, the AP as a DRL agent uses the frame collision probability to select appropriate contention window values and periodically disseminates the new CW values via beacon frames. This scheme may look similar to the proposed method in that it performs CW optimization, but different from CFX, it assumes that all devices in the network are capable of the CCOD CW optimization scheme. Recall that in this paper, we consider a network environment where legacy devices that cannot be equipped with the CFX functionalities coexist. Lee et al. proposed a backoff mechanism in which each user makes a decision on when to transmit their frames based on an observation of the channel status [19], and a similar study was conducted by Yu et al. for a situation in which users with different MAC protocols, such as Time-Division Multiple Access (TDMA) and ALOHA, coexist [18]. Kotagiri et al. utilized a distributed DRL approach to realize optimal RU selection, in which each user station locally trains its neural network on the basis of energy detection so that they can avoid possible collisions in the RU selection process [15]. Most of these schemes, however, do not fully exploit the unique features of 802.11ax, such as its TF-based protocol. In particular, unlike our CFX scheme, these approaches assume that each individual user device needs to run a DRL module, which may be practically infeasible in resource-constrained devices.

## 3. System Model

In this paper, we consider uplink transmissions in a Wi-Fi network in which $N_{c}$ CFX user devices (hereafter referred to as CFX users) and $N_{l}$ legacy user devices coexist. It was assumed that all CFX users and the AP were equipped with OFDMA functionalities. We use the term “legacy users” to refer to user devices that cannot be equipped with the CFX functionalities. Although existing 802.11ax devices are also considered legacy users by definition, this paper restricts the term “legacy users” to mean only non-802.11ax users unless otherwise stated. We also use the term “ax users” to refer to any devices that have the 802.11ax OFDMA functionalities (i.e., both CFX and 802.11ax user devices). One OFDMA frame consisted of K=9 RUs (corresponding to a 20 MHz channel) and lasted for a transmission opportunity (TXOP) duration (set as 1 ms), meaning that in a single OFDMA frame, up to 9 ax users can transmit concurrently to the uplink for 1 ms. All users, including non-ax users, were also guaranteed transmission for the same TXOP duration when they gained channel access, and in this case, they used the entire channel exclusively.

**Figure 1:**
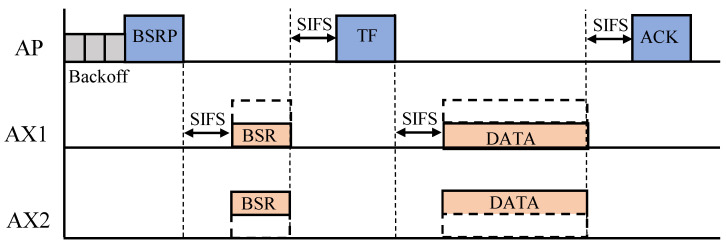
Uplink OFDMA in 802.11ax. The AP sends a BSRP frame to obtain the buffer status of the users, and they respond with BSR frames. The AP then announces the OFDMA scheduling result via a TF, and actual OFDMA data transmission begins.

As mentioned earlier, in 802.11ax, the AP coordinates simultaneous transmissions from users with the help of newly introduced types of frames, such as the TF, BSR and BSRP frames. Figure 1 shows how OFDMA transmission is performed in 802.11ax. First, the AP broadcasts a BSRP frame to the network to check how much buffered data the users are ready to send, and the users respond with a BSR frame. Based on the received buffer status reports, the AP performs OFDMA resource allocation and informs the users of the result via a TF. Following this, the users begin to transmit using the assigned RUs. We assume that the AP triggers an uplink OFDMA transmission every 10 ms. Operations related to OFDMA resource allocation are simplified throughout the paper, as mentioned earlier. More specifically, a round-robin scheduling policy is used to ensure fair access opportunities, and the number of random access RUs for UORA is fixed to three.

## 4. CFX

### 4.1. Overview

**Figure 2:**
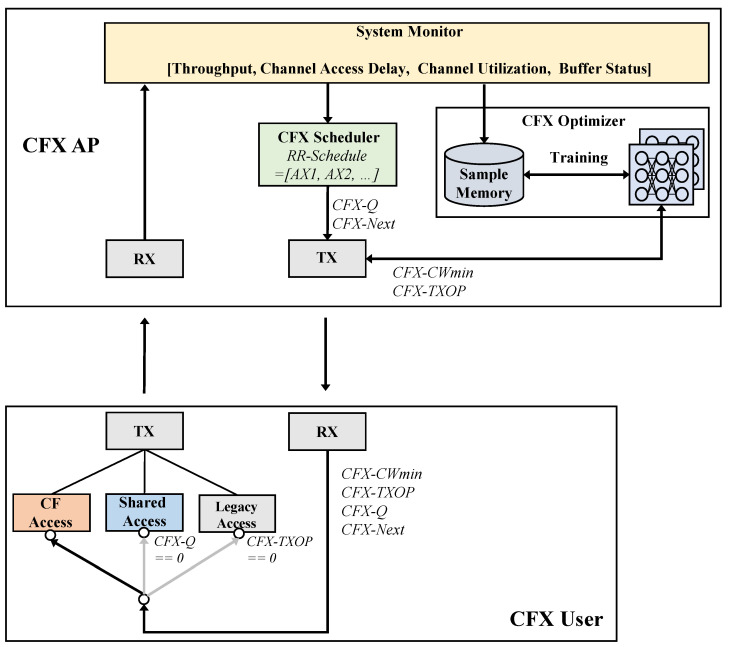
Architecture of CFX, consisting of three main components: CF channel access, shared channel access and the CFX optimizer.

In this section, we provide a brief overview of CFX. As can be seen in Figure 2, the architecture of CFX consists of three main modules: (1) CF channel access, (2) shared channel access and (3) CFX optimization.

In the first scheme, CF channel access, CFX users do not contend for the channel through the existing random access but instead are guaranteed a transmission opportunity for a certain amount of time immediately after another user’s transmission has finished. The transmission schedule for CF channel access is created and managed by the AP based on the buffer status of each user, which is tracked by the existing BSRP/BSR frame exchange protocol of 802.11ax. The second proposed scheme, based on shared channel access, extends this protocol so that the AP can obtain the buffer status of the users more effectively, and the users can access the channel more frequently. Finally, CFX optimization is conducted to maximize the performance of CFX while avoiding a situation in which the proposed scheme degrades the performance for the legacy users. In particular, we exploit an actor-critic-based PPO learning approach to control two parameters of CFX, *CFX-CWmin* and *CFX-TXOP*, thereby allowing for effective adjustment to dynamic network environments.

In the following subsections, we describe each scheme in greater detail.

### 4.2. Contention-Free Channel Access

**Figure 3:**
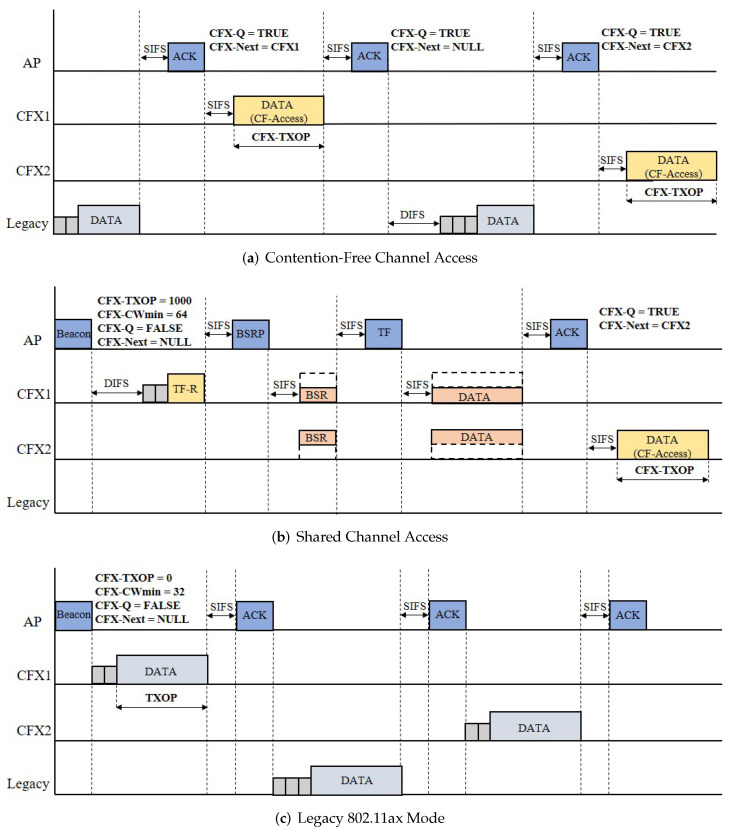
Comparison of the three access schemes. (**a**) In the CF channel access scheme, CFX users access the channel without contention. (**b**) An OFDMA multi-user transmission process can begin from CFX users through the shared channel access scheme, and (**c**) CFX can also operate in the legacy 802.11ax mode when insufficient benefits can be gained from CF channel access.

Figure 3a shows an example of CF channel access. Initially, after a legacy user’s transmission has finished, the AP designates one CFX user to transmit next via an ACK frame (specified by “CFX-Next”). In this example, user CFX1 is selected and granted permission to send his or her data after SIFS. When CF channel access is activated, the legacy user will access the channel again after the transmission by user CFX1, since CFX users do not participate in channel contention. The AP then selects one CFX user in the same way for the next CF channel access. (In this example, user CFX2 is chosen.)

When CFX users obtain a transmission opportunity through CF channel access, they can send data over the period CFX-TXOP, as shown in Figure 3a. To protect against possible frame collisions, transmission begins immediately after the SIFS interval. However, the transmission may fail for several reasons, such as a hidden user problem or poor channel conditions. Regardless of the result of the transmission, the CFX user needs to wait for the next turn once it has consumed a CF channel access opportunity.

In CFX, the AP constantly provides users with information about the CFX schedule as follows:

- CFX-TXOP: This is the maximum amount of time over which CFX users can transmit via CF channel access.
- CFX-CWmin: This is the minimum contention window value for CFX users.
- CFX-Q: This is a binary value indicating whether the buffer status of the users, as tracked by the AP, is empty or not. This value is updated each time the AP receives a frame from a user.
- CFX-Next: This is the identity (e.g., MAC address) of the CFX user granted permission to transmit. When CFX-Q is false, this value is set to NULL.

These values are broadcasted via existing control frames, such as via an ACK or a beacon, and the reserved fields of the frame header can be used for this purpose. In particular, since the values of CFX-Q and CFX-Next are determined based on the buffer status of the users and change frequently, they may need to be piggybacked on each ACK frame. The other two parameters can be fixed for a particular period of time using a control frame with a relatively long period, such as a beacon frame.

Although the proposed CF channel access has the potential to reduce the number of frame collisions, it is not always available, and in some cases, the performance of the CFX users may even be degraded. First, CF channel access is available only if CFX-Q is true. If the AP has no tracked buffer status for the users, then CFX-Q becomes false, and no further CF access can take place. In this case, the users temporarily try to access the channel without CF channel access until CFX-Q becomes valid again. The second problem occurs when the network load is light. In this case, CFX users may wait too long for other users to transmit, meaning that the channel becomes idle for unnecessarily long periods of time. To avoid this situation, we limit the maximum waiting time for CFX users to $T_{thr}$. If a channel is identified as being idle for a duration $T_{thr}$, then CFX users will access the channel via the conventional random access method. Finally, there may be some cases where CFX-TXOP is set to zero by the CFX optimization module. In this case, both CF channel access and shared channel access schemes are inactivated until CFX-TXOP takes on a valid positive value, and the system falls back to the legacy 802.11ax mode, as shown in Figure 3c.

### 4.3. Shared Channel Access

As mentioned earlier, to fully exploit the potential of CF channel access, the AP needs to track the buffer status of the users in an effective manner. Unfortunately, this is challenging, especially in a network environment with a heavy traffic load, since the AP will then rarely gain channel access. To overcome this limitation, we propose a scheme in which CFX users share their access opportunities with each other; that is, when a CFX user wins the channel contention, it passes the channel access to the AP so that the AP can start an uplink OFDMA transmission and update CFX-Q. Recall that there are some cases in which CFX users need to access the channel in a random access manner even in the CF channel access mode, as described in Section 4.2.

Figure 3b shows an example of shared channel access. When the CFX users find that CFX-Q is false, they start to contend for the channel in a random access manner. In the example given here, user CFX1 wins the contention, and this sends the AP a short control frame similar to a TF, which we call TF-Request (TF-R). Upon receiving it, the AP begins an OFDMA transmission as if it were the original winner of the channel contention. In this way, the AP can update CFX-Q and resume CF channel access (user CFX2 in the figure).

One notable feature of the proposed scheme is that OFDMA multi-user transmissions can begin from CFX users. However, this does not necessarily mean that only CFX users can participate in this type of OFMDA transmission. Since after TF-R frame transmission the existing BSRP/BSR exchange protocol starts, through this protocol legacy 802.11ax, users also can participate in OFDMA transmission. They just do not understand the new frame TF-R; that is, they cannot send TF-R frames and cannot use the contention-free channel access, but except for these instances, there are no restrictions on accessing the channel.

In addition to allowing the AP to obtain the buffer status of the ax users effectively, the shared channel access scheme offers additional benefits for the system. First, ax users can access the channel more frequently, since OFDMA transmissions can be activated more often. This helps to reduce the channel access delay for the ax users. Secondly, in the shared channel access scheme, CFX users send a short control frame (i.e., TF-R), meaning that the time loss due to collision could be small, especially when it collides with other control frames.

### 4.4. CFX Optimization

Through the use of the two CFX access schemes described above, we can effectively lower the probability of frame collision. However, if these benefits are not properly shared with all of the users in the system, including non-CFX users, several unexpected issues may arise. For example, in the shared channel access scheme, the use of an additional control frame (i.e., TF-R) introduces an overhead, albeit a small one, into the system. One might think that CFX users would rather lose their transmission opportunities due to the sharing, which could lead to performance degradation. For example, in Figure 3b, user CFX1 shares its transmission opportunity despite being able to use the channel exclusively. Meanwhile, the legacy users, in particular non-802.11ax users, may experience a longer access delay as the CFX users access the channel more frequently. For example, in Figure 3a, the legacy user needs to wait until user CFX1’s transmission is finished, resulting in a longer channel access delay. To tackle these issues, we optimize two key parameters of CFX—CFX-CWmin and CFX-TXOP—with DRL, which will be described in the following subsections.

#### 4.4.1. Problem Formulation

We view the network as a time-slotted system, where each slot corresponds to one beacon interval. At the beginning of each slot *t*, the AP determines the values of CFX-CWmin and CFX-TXOP. Once these are fixed, they persist for the entire slot duration and are determined again when the next slot begins. Our goal is to adaptively tune these two parameters, denoted by $A=\{a_{t}:t=0,1,\ldots ,T_{max}\}$, to maximize the total throughput of the users, under the constraint that the performance requirements of each user are met, as follows:

(1) $$\begin{array}{c} P\text{:}\;\underset{a_{t}\in A,\forall t}{\mathrm{maximize}\;}\sum_{t=0}^{T_{max}}\left(s_{t}^{c}+s_{t}^{l}\right) \end{array}$$

(2) $$\begin{array}{c} C1\text{:}\;\left(S^{c},S^{l}\right)\leq \left(s_{t}^{c},s_{t}^{l}\right) \end{array}$$

(3) $$\begin{array}{c} C2\text{:}\;\left(d_{t}^{c},d_{t}^{l}\right)\leq \left(D^{c},D^{l}\right) \end{array}$$

(4) $$\begin{array}{c} C3\text{:}\;\left(s_{t}^{c},s_{t}^{l},d_{t}^{c},d_{t}^{l}\right)=f\left(a_{t}\right), \end{array}$$

where $s_{t}^{c}$ and $s_{t}^{l}$ represent the average throughputs of the CFX users and the legacy users at slot *t*, respectively, and $d_{t}^{c}$ and $d_{t}^{l}$ represent the average access delays. The constraints in Equations (2) and (3) indicate the minimum throughput and the maximum channel access delay requirements for both user types, where $S^{c}$ and $S^{l}$ represent the minimum throughputs for CFX and legacy users, respectively, and $D^{c}$ and $D^{l}$ are the values of the maximum channel access delay. In the last constraint, we define a function *f* that takes action $a_{t}$ and returns the average throughput and access delay of the users.

Although problem P looks simple, it is in fact non-trivial to solve. First, this problem is combinatorial, requiring an exhaustive search to find the optimal solution, which is practically infeasible. Another difficulty in solving this problem lies in the nonlinearity of the function *f*. In addition, the function *f* is actually characterized by numerous system elements, such as the number of users, the length of TXOP, the frame arrival rates and so on, some of which change randomly over time, making it hard for the AP to predict these in advance. Recall that the AP can only use partial observable information about the network.

#### 4.4.2. Deep Reinforcement Learning Model

To solve the optimization problem P, we exploit a DRL approach in which an agent learns from interactions with its environment [35]. In this approach, the agent takes an action based on observations of its state, and depending on this action, it obtains some reward. Reinforcement learning is used to train the agent to generate the optimal policy in which the cumulative reward is maximized.

In the following, we first introduce the main parameters of the proposed DRL model and briefly explain the learning algorithm used in this work. Note that since the term “step” is commonly used in reinforcement learning, we will use the terms “slot” and “step” interchangeably. In addition, we omit the subscript *t* from some parameter expressions for simplicity of notation.

##### Action

The action space per system state $a_{t}$ is defined as a set of two values, denoted as ($a_{t}^{cw}$, $a_{t}^{txop}$), each of which indicates a direction of movement along the dimensions of the CFX-CWmin and CFX-TXOP search spaces. More specifically, each action has three possible values: −1, 0 and 1. The two parameters are updated by the following rule:

(5) $$\begin{array}{c} \mathrm{CFX}-\mathrm{CWmin}:=\mathrm{CFX}-\mathrm{CWmin}\times 2^{a_{t}^{cw}} \end{array}$$

(6) $$\begin{array}{c} \mathrm{CFX}-\mathrm{TXOP}:=\mathrm{CFX}-\mathrm{TXOP}+w\times a_{t}^{txop}, \end{array}$$

where *w* is the step size. Note that CFX-CWmin and CFX-TXOP are limited to their respective ranges; if the updated value is out of range, then the old value will be retained.

**Figure 4:**
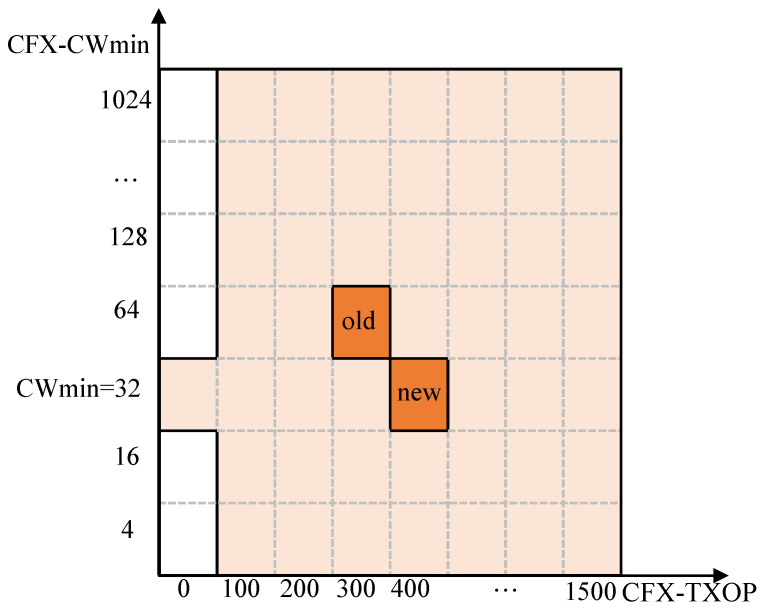
Example of the action space. The agent can move along the dimensions of the CFX-CWmin and CFX-TXOP search spaces. The action (−1, 1) at the position marked “old” will move the agent to the position marked “new”, corresponding to a CFX-CWmin of 32 and a CFX-TXOP of 400 ms.

Figure 4 shows an example of the action space, where the minimum and maximum CFX-CWmin are 4 and 1024, respectively, and the CFX-TXOP values range from 0 to 1500 ms. In the figure, each shaded small box represents a valid action that can be taken. Note that when CFX-TXOP is set to zero, CFX simply returns to the legacy 802.11ax scheme, and thus CFX-CWmin is also set to the original value of CWmin.

##### State

We define the state of the system at slot *t*, $o_{t}$ as the following tuple of three vectors:

(7) $$\begin{array}{c} o_{t}=\left({\vec{n}}_{t},{\vec{a}}_{t},{\vec{v}}_{t}\right), \end{array}$$

where the first vector ${\vec{n}}_{t}$ represents the number of each type of user at step *t*. The vector ${\vec{a}}_{t}$ represents the two control parameters used (i.e., ${\vec{a}}_{t}={\left[\mathrm{CFX}-\mathrm{CWmin},\mathrm{CFX}-\mathrm{TXOP}\right]}^{⊺}$). The last vector represents the vector of the channel utilization for slot *t*, which has the following four elements: ${\vec{v}}_{t}={\left[v^{CFX},v^{LE},v^{COL},v^{IDLE}\right]}^{⊺}$, each of which denotes the ratio of the time occupied by CFX users, legacy users, frame collisions and an idle state, respectively, to the total duration of slot *t*. Note that we can actually exclude one of these four elements from ${\vec{v}}_{t}$, since it is just a linear combination of the other three values (i.e., $v^{CFX}+v^{LE}+v^{COL}+v^{IDLE}=1$) and is not helpful to the learning process. All of these values are normalized to the range [0, 1].

##### Reward

We use the same objective function as in problem P; in other words, we use the total throughput of the users as the reward for the pair of items consisting of action $a_{t}$ and state $o_{t}$. We denote this as $r_{t}$. In addition, to ensure the minimum performance requirements, we set the reward to zero as a penalty if Equations (2) and (3) are not satisfied.

#### 4.4.3. Deep Reinforcement Learning Algorithm

As mentioned earlier, the goal of the reinforcement learning algorithm is to train the agent to generate the optimal policy that maximizes the cumulative reward *G*, defined as the sum of the current reward and the discounted future reward, as follows:

(8) $$\begin{array}{c} G_{t}=\sum_{k=t}^{B}\gamma^{k-t}r_{k}, \end{array}$$

where γ is a discount factor between zero and one, *B* is the number of steps in one episode and $r_{k}$ is the reward at step *k*. In practice, Equation (8) represents the expected reward starting from slot *t*, which will be used later.

In this work, we adopt an actor-critic-based DRL approach. The actor, denoted as $\pi_{\theta }\left(a_{t};o_{t}\right)$, where θ represents its parameters, performs the task of learning what action to take given $o_{t}$ and outputs the conditional probability of taking each action $a_{t}$ when in a state $o_{t}$. The critic, denoted as $V_{\phi }\left(o_{t}\right)$, where ϕ represents its parameters, makes an observation $o_{t}$ and returns the corresponding expectation value of the discounted long-term reward (called the value function) to evaluate whether the action taken by the actor leads the system to a better state or not. By comparing the rating values of the current policy and a new policy, the actor decides how he or she wants to improve him or herself to take better actions.

**Figure 5:**
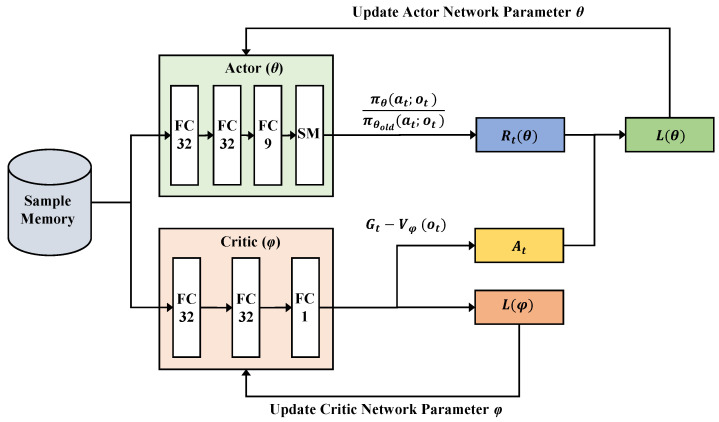
Actor-critic based PPO. The actor and critic networks are trained to complement each other.

Figure 5 illustrates the two network models used in this paper. The actor network consists of three fully connected (FC) layers with ReLU activation, and a softmax layer is added at the end of the network. Note that the last FC layer has a size of nine, since the total number of possible actions in the DRL model is nine (three actions for each parameter). The structure of the critic network is similar to that of the actor, except that it ends with an FC layer of a size of one. (Recall that it only outputs a single evaluation value.)

To train the two networks, we employ the PPO learning algorithm [36], and we briefly summarize how it works. PPO is a policy-based algorithm that learns the optimal policy π directly without computing the Q value, a metric that indicates the goodness of the selected state/action pair. One important feature of this algorithm is that to achieve efficient training, it attempts to make a large policy update while ensuring that the new policy ($\pi_{\theta }$) does not vary a great deal from the old one ($\pi_{\theta_{old}}$), an approach that is referred to as a trust region method. In PPO, a trust region $R_{t}\left(\theta \right)$ is defined as the probability ratio of the new policy to the old policy as follows:

(9) $$\begin{array}{c} R_{t}\left(\theta \right)=\frac{\pi_{\theta }\left(a_{t};o_{t}\right)}{\pi_{\theta_{old}}\left(a_{t};o_{t}\right)} \end{array}$$

Using $R_{t}\left(\theta \right)$, the objective functions used to update θ and ϕ for the two networks are defined as follows:

(10) $$\begin{array}{c} L\left(\theta \right)=E[\min\left(R_{t}\left(\theta \right)A_{t},g_{\epsilon }\left(R_{t}\left(\theta \right)A_{t}\right)\right)] \end{array}$$

(11) $$\begin{array}{c} L\left(\phi \right)=E[{\left(G_{t}-V_{\phi }\left(o_{t}\right)\right)}^{2}], \end{array}$$

where $A_{t}$ is called the advantage and is the difference between the expected total reward $G_{t}$ in Equation (8) and the value function $V_{\phi }\left(o_{t}\right)$ obtained from the critic network. The function $g_{\epsilon }$ is a clipping function that truncates the ratio $R_{t}\left(\theta \right)$ to the range [1−ϵ,1+ϵ]. The parameter ϵ is used to ensure that the policy updates are within the trust region. In this paper, we use a value of 0.2 for ϵ. It is worthwhile to note that although to make the learning algorithm more sample-efficient, some approaches such as Actor Critic with Experience Replay (ACER) [37] exploit a replay buffer, in general, most on-policy algorithms such as PPO do not need to use it because updates happen within small-sized batches. The sample memory denoted in Figure 5 is actually used to just hold previous experiences and handle mini-batches in training.

The overall PPO learning algorithm is summarized in Algorithm 1. We conclude this subsection by giving two major reasons for our choice of PPO. First, the PPO learning algorithm is suitable for operation in the continuous environment (i.e., $o_{t}$) of the proposed DRL model, and we can take advantage of both the value-based and policy-based methods. Secondly, we can find optimal solutions at a lower computational cost compared with the trust region policy optimizer (TRPO), another widely used trust region-based learning algorithm that can provide better learning stability [38].
**Algorithm 1 **PPO Learning Algorithm1:Initialize θ and ϕ with random numbers2:**for** each iteration **do**3:    Collect *B* number of trajectories following the policy $\pi_{\theta }$ in the actor4:    Update θ as $\theta +\alpha {▿}_{\theta }L\left(\theta \right)$ using Equation (10).5:    Update ϕ as $\phi +\alpha {▿}_{\phi }L\left(\phi \right)$ using Equation (11).6:**end for**

### 4.5. Summary

The pseudocode for the two proposed algorithms for slot *t* is given in Algorithms 2 and 3 from the perspectives of the AP and the CFX users, respectively.

The complexity of CFX comes mainly from two factors: TF-R and the deep learning module. First, since TF-R frames do not need to carry much information, they typically require fewer bytes than the existing trigger frame of 802.11ax, which requires at least 30 bytes to convey OFDMA scheduling information for multiple users [1]. As will be seen later, this overhead is almost negligible thanks to the performance gains of CFX. Second, in the proposed scheme, the AP needs additional computation to execute the CFX optimization module, and similar architectures are widely accepted in various recently proposed schemes [14,17]. In addition, as all deep learning related operations of CFX run only on the AP as shown in Algorithms 2 and 3, there is no additional burden on the user devices.

It is well known that the learning algorithm performance is affected by multiple factors, such as model complexity, episode length, mini-batch size and learning rate, and in particular, it also depends on how the action space and the state are defined. In this study, we propose a small discrete action space (nine actions in total), but this design has limitations in that parameter changes must always be made in small steps, and the system state (i.e., Equation (7)) becomes unnecessarily large. We leave further study of the performance of CFX according to various deep learning models and algorithms as future work.
**Algorithm 2 **CFX-AP 1:**if** at the beginning of slot *t* **then** 2:    Obtain $o_{t}$, and select $a_{t}$ using the policy in the actor 3:    Store the experience ($o_{t-1}$, $a_{t-1}$, $r_{t-1}$, $o_{t}$) in the memory 4:    Update CFX-CWmin and CFX-TXOP using Equations (5) and (6) 5:    **if** CFX-TXOP == 0 **then** 6:        Enter the legacy 802.11ax mode 7:        Set CFX-CWmin to CWmin 8:    **end if** 9:    Broadcast CFX-TXOP and CFX-CWmin10:**end if**11:**while** slot *t* is not finished **do**12:    **if** data frame received **then**13:        Update CFX-Q14:    **end if**15:    **if** ack to transmit **then**16:        **if** CFX-TXOP > 0 and CFX-Q is true **then**17:           Set CFX-Next by selecting one ax user18:        **else**19:           Set CFX-Next to NULL20:        **end if**21:        Send the ack frame with CFX-Q and CFX-Next piggybacked.22:    **end if**23:**end while**

**Algorithm 3 **CFX-User
 1:**if** CFX-CWMin and CFX-TXOP received **then** 2:    Update CFX-CWMin and CFX-TXOP 3:    **if** CFX-TXOP == 0 **then** 4:        Enter the legacy 802.11ax mode 5:    **end if** 6:
**end if**
 7:**while** slot *t* is not finished **do** 8:    **if** ack received **then** 9:        **if** CFX-Q is false **then**10:           shared channel access11:        **else if** CFX-Next matches **then**12:           contention-free channel access13:        **end if**14:    **end if**15:
**end while**



## 5. Performance Evaluation

### 5.1. Settings

In this section, the performance of the proposed scheme is evaluated. The core functionalities of CFX (i.e., CF channel access, shared channel access and CFX optimization) are implemented on top of the MATLAB deep learning framework (the code can be found at this link (accessed on 21 November 2022: https://sites.google.com/view/cilab-dku/research) [20]. We set up a 200 × 200 m grid topology and randomly place $N_{c}$ CFX devices and $N_{l}$ non-802.11ax devices on it. For all user devices, data frames of a size of 1500 B arrive in the buffer following an exponential distribution with a rate of λ. Out of *K* subchannels, three subchannels are allocated for random access RUs, and the minimum OCW and maximum OCW values are set as 4 and 32, respectively. The total simulation time is set to 10 min, and we repeat each simulation 100 times and take the average values for evaluation.

For the deep learning module of CFX, the lengths of one episode and one step are set to 10 s and 200 ms, respectively. The two neural networks are trained with the Adam optimizer [39] with a learning rate of 1 × 10${}^{-3}$on an Intel-i7 machine with 16 GB RAM and an NVIDIA RTX 3080 GPU. The requirement parameters in Equations (2) and (3) are set based on the performance measurement of the legacy 802.11ax scheme. The mini-batch size and the discount factor γ are set to 128 and 0.99, respectively, and the L2 regularizer is applied with a regularization factor of 1 × 10${}^{-4}$. The other key parameters used in the evaluations are given in Table 1.

We compare the performance of CFX with those of the legacy 802.11ax mode, CFX without optimization (denoted as CFX w/o opt), and CCOD with DQN [17]. Note that in legacy 802.11ax and CCOD, $N_{c}$ CFX users act as legacy 802.11ax users. When implementing CCOD, we have to make several modifications to the original CCOD to ensure a fair comparison. First, different from the other schemes used in this evaluation, OFDMA multi-user transmission is not considered in CCOD. To create a similar test environment, we implement CCOD on top of the same OFDMA-enabled 802.11ax MAC used in the other approaches. Second, CCOD assumes that all nodes in the network can use the contention window optimization method. This is not actually consistent with our assumption that legacy user devices cannot be modified, but to better investigate the impact of CW values on performance, we allow them to adjust their CW values via CCOD.

### 5.2. Overall Results

**Figure 6:**
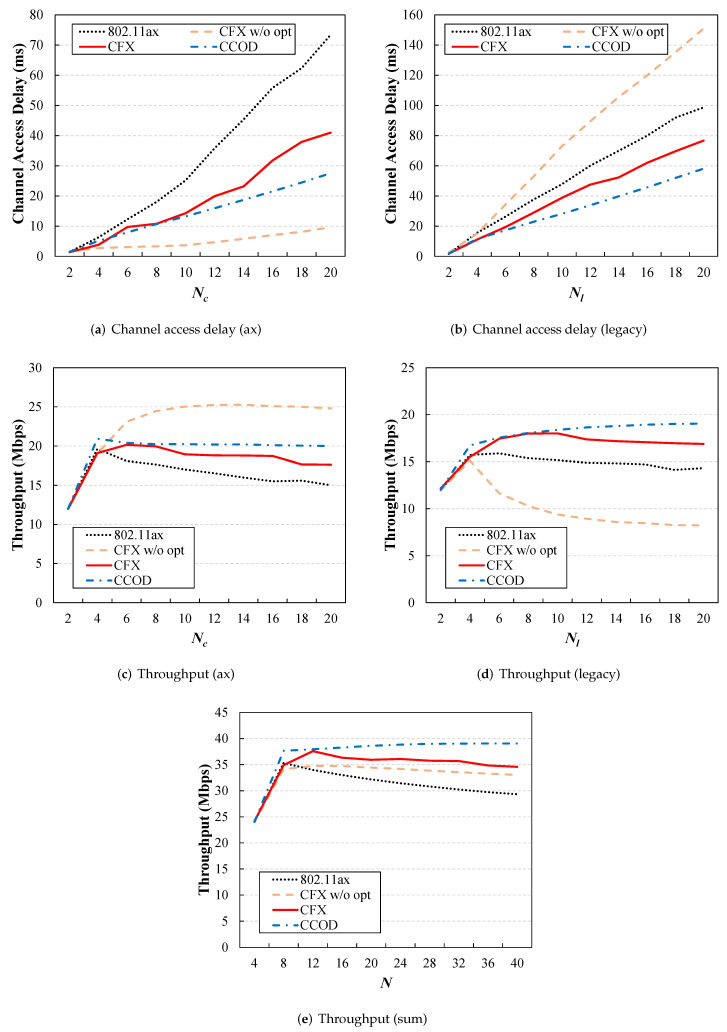
Throughput and channel access delay for the four schemes as a function of *N*. CFX achieved higher performance than the conventional 802.11ax in terms of both total throughput (**c**–**e**) and channel access delay (**a**,**b**). When optimization was not applied, CFX tended to work favorably only for ax users, causing an unfairness problem.

Figure 6 compares the performance of the four schemes in terms of overall throughput and channel access delay for varying numbers of users ($N=N_{c}+N_{l}$). From the results, we can see that CFX achieved an improvement in the maximum throughput of 5% over the legacy 802.11ax. For both the ax users and legacy users, the channel access delay was reduced by more than 30 ms, and CFX was found to be consistently effective even in dense network scenarios.

**Figure 7:**
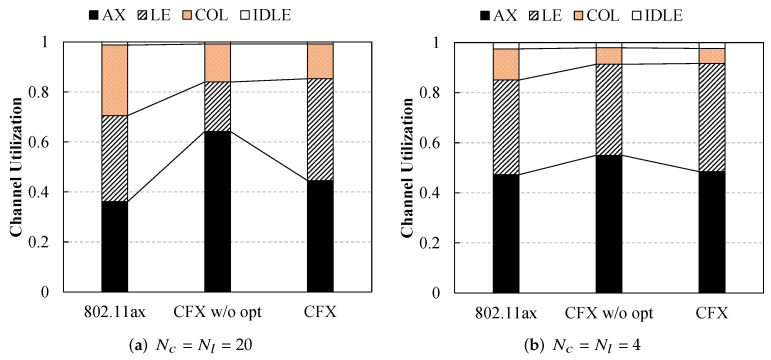
Channel utilization. CFX effectively reduced the probability of frame collision and thus achieved better performance for both ax and legacy users. This result is more clearly observed when the network is busy.

This is clearly illustrated in Figure 7, which presents a plot of the channel utilization for the three schemes. When N=40, the frame collision time made up about 28% of the total channel time for the legacy 802.11ax scheme. However, this dropped significantly to 14% with the proposed method (Figure 7a). Similarly, when there were moderate numbers of users, this proportion was also effectively reduced from 12% to 6% with CFX (Figure 7b).

The savings from the reduced frame collisions mean that there were more transmission opportunities for the users, but the performance of the system varied depending on how these were allocated. In particular, from Figure 6b,d, we can see that when optimization was not applied, CFX (i.e., CFX without optimization) worked favorably only for the ax users, and as a result, the performance for the existing legacy users was worse than that for 802.11ax. This limitation was addressed by the use of PPO-based optimization. In this case, both the ax and the legacy users had more transmission opportunities, indicating that the benefits from the reduced collisions were fairly shared with all users in the network.

It is a well-known fact that the contention window value has a great impact on the performance of the MAC protocol in 802.11 systems [12], which is also clearly observed in the CCOD evaluation results in Figure 6. It seems that CCOD outperformed CFX, but this was only because CCOD’s CW value optimization technique was applied not only to the ax users but also to the legacy users. On the other hand, the proposed method can achieve performance not significantly different from that of CCOD without changing the behavior of the legacy users.

### 5.3. Network Dynamics

One design principle of CFX is that it should appropriately adjust to changes in the network status. To investigate this aspect, we started a simulation with four users (two of each type) and then increased this to 20 (10 of each type) in the middle of the simulation. During the simulation, we recorded the changes in the values of CFX-CWmin and CFX-TXOP.

**Figure 8:**
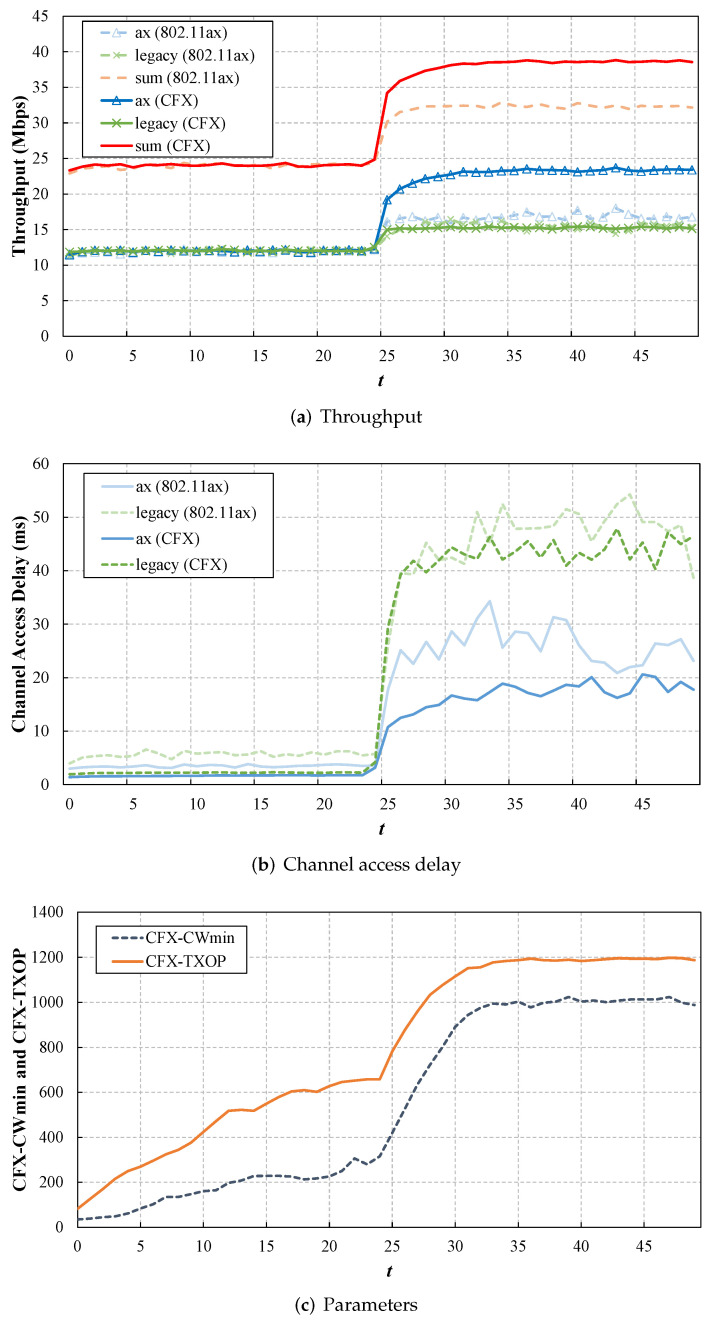
CFX according to varying network status. We increased the number of users from 4 to 20 at t=25. At first, the agent tended to use the access schemes of CFX less often, but as the access probability increased, it achieves higher values for CFX-CWmin and CFX-TXOP.

Figure 8 shows the throughput and the channel access delay of the users, as well as the values of CFX-CWmin and CFX-TXOP, as a function of *t*. From the results, we can see that CFX effectively adjusted the two parameters in response to the network changes. In the first half of the simulation, when the network load was relatively light, CFX tended to take on low CFX-CWmin and CFX-TXOP values, which implies that it rarely used the CF and shared access schemes, since the expected performance gain through CFX was not large compared with the overhead. In particular, at the very beginning, we can observe that CFX frequently set CFX-TXOP to zero and operated in the legacy 802.11ax random access mode. However, as the amount of data to transmit continued to accumulate in the buffer for each user, the channel access probability hence gradually increased. We can clearly see that CFX tended to achieve higher CFX-CWmin and CFX-TXOP values, where the value of CFX-CWmin increased to avoid unnecessary frame collisions, and at the same time, a large CFX-TXOP value was generated to maximize the benefits from the CF channel access.

### 5.4. Homogeneous 802.11ax Networks

**Figure 9:**
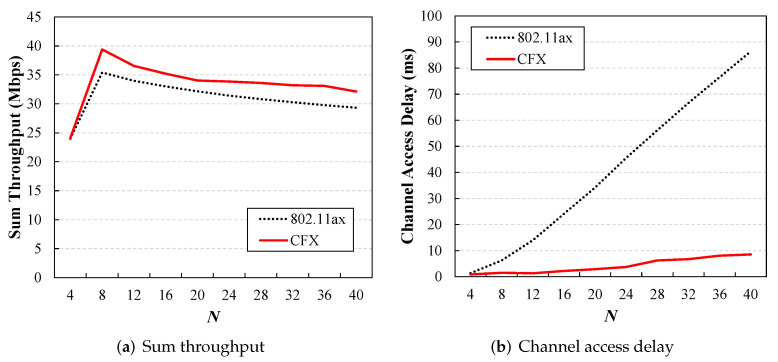
Performance of CFX in homogeneous networks. The users obtained about 4 Mbps more throughput in CFX on average than in the legacy 802.11ax mode (**a**), and at the same time, the channel access delay was reduced to 10 ms from 85 ms when the number of users was 40 (**b**).

Thus far, we have investigated the performance of CFX in networks where 802.11ax and non-802.11ax coexist. In the following, we measure the performance of CFX in homogeneous networks containing only ax users. Figure 9 shows the sum throughput and the channel access delay of the legacy 802.11ax and CFX schemes for numbers of users varying from 4 to 40. It can be seen that in general, CFX outperformed the legacy 802.11ax scheme. In CFX, the users obtained a throughput that was about 4 Mbps higher on average than that in the legacy 802.11ax mode. In particular, the channel access delay was greatly reduced in CFX. Even when the number of users was 40, the average access delay for the ax users was only 10 ms, whereas it increased to 85 ms in the legacy 802.11ax mode. These results are consistent with our motivating assumption that OFDMA uplink transmissions invoked by the AP become less effective when the number of contending users is too high.

From the channel access delay results for the 802.11ax scheme shown in Figure 6, we can see that the ax users had a longer access delay in a homogeneous network. For example, when $N_{c}=N_{l}=20$, the access delay of the ax users in 802.11ax was about 73 ms (Figure 6a), while for $N_{c}=40$, it was 85 ms (Figure 6b). This result was due to the size of *K*. The main mechanism used to reduce the access delay involved serving multiple users at once via OFDMA, but if *K* was too small, only a limited number of users could benefit from one OFDMA transmission. In this evaluation, *K* was set to 9, which may be sufficient to serve 20 ax users in a network where 802.11ax and non-802.11ax users coexist but is somewhat insufficient to serve 40 ax users in a homogeneous network.

## 6. Conclusions

In this paper, we propose a new channel access scheme for 802.11ax, called CFX, based on CF channel access and shared channel access, which significantly reduces the frame collisions in Wi-Fi. In order to appropriately utilize the savings from the reduced frame collisions, we employed an actor-critic PPO-based DRL approach in the design of CFX. The results of an extensive evaluation show that CFX not only greatly improved the uplink performance of Wi-Fi in terms of throughput and channel access delay but also dynamically adjusted its parameters in response to the changes in the network status.

## Figures and Tables

**Table 1:** Default simulation parameters

Parameter	Value
CWmin	32
CWmax	1024
TXOP	1 ms
λ	500 frames/s
frame size	1500 B
data rate	54 Mbps
$N_{c}=N_{l}$	10
*K*	9
OCWmin	4
OCWmax	32
$T_{thr}$	320 μs
*w*	100 ms
$S^{c}$	16 Mbps
$S^{l}$	16 Mbps
$D^{c}$	15 ms
$D^{l}$	40 ms

## Data Availability

Not applicable.

## References

[1] (2017). *P802.11ax*; IEEE Draft Standard for Information Technology—Telecommunications and Information Exchange Between Systems Local and Metropolitan Area Networks—Specific Requirements Part 11: Wireless LAN Medium Access Control (MAC) and Physical Layer (PHY) Specifications Amendment Enhancements for High Efficiency WLAN.

[2] de Vegt R. Reduced latency benefits of Wi-Fi 6 OFDMA. The Beacon Blog, Wi-Fi.org 2021. https://www.wi-fi.org/beacon/rolf-de-vegt/reduced-latency-benefits-of-wi-fi-6-ofdma/.

[3] Shariatmadari H., Ratasuk R., Iraji S., Laya A., Taleb T., Jäntti R., Ghosh A. (2015). Machine-type Communications: Current Status and Future Perspectives Toward 5G Systems. IEEE Commun. Mag..

[4] Ogudo K.A., Muwawa Jean Nestor D., Ibrahim Khalaf O., Daei Kasmaei H. (2019). A Device Performance and Data Analytics Concept for Smartphones’ IoT Services and Machine-Type Communication in Cellular Networks. Symmetry.

[5] Chen X., Li Z., Chen Y., Wang X. (2019). Performance Analysis and Uplink Scheduling for QoS-Aware NB-IoT Networks in Mobile Computing. IEEE Access.

[6] Lee K.H. (2019). Performance Analysis of the IEEE 802.11ax MAC Protocol for Heterogeneous Wi-Fi Networks in Non-Saturated Conditions. Sensors.

[7] Carpenter T. 802.11 Beacon Intervals. https://www.cwnp.com/cwnp-wifi-blog/80211-beacon-intervals/.

[8] 7SIGNAL. Boosting WI-FI Performance with Beacon and Beacon Interval. https://www.7signal.com/news/blog/controlling-beacons-boosts-wi-fi-performance.

[9] Shih K.P., Liao W.H., Chen H.C., Chou C.M. (2009). On avoiding RTS collisions for IEEE 802.11-based wireless ad hoc networks. Comput. Commun..

[10] Nordin N., Dressler F. Effects and implications of beacon collisions in co-located IEEE 802.15. 4 networks. Proceedings of the 2012 IEEE Vehicular Technology Conference (VTC Fall).

[11] Bankov D., Khorov E., Lyakhov A., Schelstraete S. Beacons in dense Wi-Fi networks: How to befriend with neighbors in the 5G world?. Proceedings of the 2016 IEEE 17th International Symposium on A World of Wireless, Mobile and Multimedia Networks (WoWMoM).

[12] Bianchi G. (2000). Performance Analysis of the IEEE 802.11 Distributed Coordination Function. IEEE J. Sel. Areas Commun..

[13] Bellalta B., Kosek-Szott K. (2019). AP-initiated multi-user transmissions in IEEE 802.11 ax WLANs. Ad. Hoc. Netw..

[14] Han M., Chen Z., Cai L.X., Luan T.H., Hou F. A Deep Reinforcement learning based Approach for Channel Aggregation in IEEE 802.11 ax. Proceedings of the GLOBECOM 2020—2020 IEEE Global Communications Conference.

[15] Kotagiri D., Nihei K., Li T. Distributed Convolutional Deep Reinforcement Learning based OFDMA MAC for 802.11ax. Proceedings of the ICC 2021—IEEE International Conference on Communications.

[16] Wu C.M., Kao Y.C., Chang K.F., Tsai C.T., Hou C.C. (2021). A Q-Learning-Based Adaptive MAC Protocol for Internet of Things Networks. IEEE Access.

[17] Wydmański W., Szott S. Contention Window Optimization in IEEE 802.11ax Networks with Deep Reinforcement Learning. Proceedings of the 2021 IEEE Wireless Communications and Networking Conference (WCNC).

[18] Yu Y., Wang T., Liew S.C. (2019). Deep-Reinforcement Learning Multiple Access for Heterogeneous Wireless Networks. IEEE J. Sel. Areas Commun..

[19] Lee C.K., Hyong Rhee S. Collision Avoidance in IEEE 802.11 DCF using a Reinforcement Learning Method. Proceedings of the 2020 International Conference on Information and Communication Technology Convergence (ICTC).

[20] Mathworks (2020). MATLAB Deep Learning Toolbox.

[21] Karthik R.M., Palaniswamy S. Resource Unit (RU) based OFDMA Scheduling in IEEE 802.11ax System. Proceedings of the 2018 International Conference on Advances in Computing, Communications and Informatics (ICACCI).

[22] Dovelos K., Bellalta B. (2019). Optimal Resource Allocation in IEEE 802.11ax Uplink OFDMA with Scheduled Access. arXiv.

[23] Wang K., Psounis K. Scheduling and Resource Allocation in 802.11ax. Proceedings of the IEEE INFOCOM 2018—IEEE Conference on Computer Communications.

[24] Bankov D., Didenko A., Khorov E., Lyakhov A. OFDMA Uplink Scheduling in IEEE 802.11ax Networks. Proceedings of the 2018 IEEE International Conference on Communications (ICC).

[25] Lanante L., Uwai H.O.T., Nagao Y., Kurosaki M., Ghosh C. Performance Analysis of the 802.11ax UL OFDMA Random Access Protocol in Dense Networks. Proceedings of the 2017 IEEE International Conference on Communications (ICC).

[26] Naik G., Bhattarai S., Park J. Performance Analysis of Uplink Multi-User OFDMA in IEEE 802.11ax. Proceedings of the 2018 IEEE International Conference on Communications (ICC).

[27] Lee J. OFDMA-based Hybrid Channel Access for IEEE 802.11ax WLAN. Proceedings of the 2018 14th International Wireless Communications Mobile Computing Conference (IWCMC).

[28] Kosek-Szott K., Domino K. (2022). An Efficient Backoff Procedure for IEEE 802.11ax Uplink OFDMA-Based Random Access. IEEE Access.

[29] Khorov E., Loginov V., Lyakhov A. Several EDCA Parameter Sets for Improving Channel Access in IEEE 802.11ax Networks. Proceedings of the 2016 International Symposium on Wireless Communication Systems (ISWCS).

[30] Lee K.H. (2019). Using OFDMA for MU-MIMO User Selection in 802.11ax-Based Wi-Fi Networks. IEEE Access.

[31] Kim Y., Kim G., Lee J., Choi W. UL-MU Transmissions in IEEE 802.11ax Networks. Proceedings of the 2020 IEEE International Conference on Consumer Electronics (ICCE).

[32] Chen S.C., Li C.Y., Chiu C.H. An Experience Driven Design for IEEE 802.11ac Rate Adaptation based on Reinforcement Learning. Proceedings of the IEEE INFOCOM 2021—IEEE Conference on Computer Communications.

[33] Peserico G., Fedullo T., Morato A., Vitturi S., Tramarin F. Rate Adaptation by Reinforcement Learning for Wi-Fi Industrial Networks. Proceedings of the 2020 25th IEEE International Conference on Emerging Technologies and Factory Automation (ETFA).

[34] (2013). IEEE Standard for Information Technology–Telecommunications and Information Exchange between Systems Local and Metropolitan Area Networks–Specific Requirements–Part 11: Wireless LAN Medium Access Control (MAC) and Physical Layer (PHY) Specifications–Amendment 4: Enhancements for Very High Throughput for Operation in Bands below 6 GHz. 802.11ac-2013.

[35] Sutton R.S., Barto A.G. (2018). Reinforcement Learning: An Introduction.

[36] Schulman J., Wolski F., Dhariwal P., Radford A., Klimov O. (2017). Proximal Policy Optimization Algorithms. arXiv.

[37] Wang Z., Bapst V., Heess N., Mnih V., Munos R., Kavukcuoglu K., de Freitas N. (2016). Sample efficient actor-critic with experience replay. arXiv.

[38] Schulman J., Levine S., Abbeel P., Jordan M., Moritz P. Trust Region Policy Optimization. Proceedings of the International Conference on Machine Learning.

[39] Kingma D.P., Ba J. (2014). Adam: A Method for Stochastic Optimization. arXiv.

